# Malignant hyperthermia when dantrolene is not readily available

**DOI:** 10.1186/s12871-021-01328-3

**Published:** 2021-04-16

**Authors:** Xiaodan Gong

**Affiliations:** 1 Department of Cardiology, Charité – Universitätsmedizin Berlin, corporate member of Freie Universität Berlin and Humboldt-Universität zu Berlin, Charité University Medicine, Campus Virchow Klinikum, Augustenburger Platz 1, 13353 Berlin, Germany; 2 Department of Anesthesiology, The Second Clinical Medical College, Yangtze University, Jingzhou, 434020 China

**Keywords:** Malignant hyperthermia, Dantrolene, Mortality, Enzyme, Treatment

## Abstract

**Background:**

Malignant hyperthermia is a rare but life-threatening pharmacogenetic muscle disorder characterized by abnormal hypermetabolic reactions and commonly triggered in susceptible individuals by volatile anesthetics or succinylcholine, or both. Unfortunately, the specific medicine dantrolene is not readily available in many countries including China. The aim of this study was to find the characteristics of malignant hyperthermia under the situation that dantrolene is not readily available.

**Methods:**

The cases of malignant hyperthermia reported on the most commonly used databases in China from 1985 to 2020 were analyzed. The inclusion criteria were the MH episodes only related to anesthesia. The exclusion criteria were dubious MH episodes only caused by Ketamine administration or MH episodes irrelevant to anesthesia. Independent samples t-test and Pearson’s chi-squared test were applied to assess the difference between the survived and death cases.

**Results:**

Ninety-two cases of malignant hyperthermia reported on the most commonly used databases in China from 1985 to 2020 were analyzed. Median (IQR [range]) age was 18.5 (11.8–37.0 [0–70.0]) years. Compared with the survived cases, the death cases had higher maximum end-tidal partial pressure of CO_2_ (*P* = 0.033), the maximum arterial partial pressure of CO2 (*P* = 0.006), temperature first measured when the patient was first discovered abnormal (*P* = 0.012), and maximum temperature (*P* < 0.001). Besides, the death cases had less minimum pH (*P* < 0.001) and higher potassium (*P* < 0.001) and were more likely to have coagulation disorders (*p* = 0.018). Concerning treatment, cases used furosemide (*P* = 0.024), mannitol (*P* = 0.029), blood purification treatment (*P* = 0.017) had the advantage on the outcome. Creatine phosphokinase, myoglobin, and MB isoenzyme of creatine phosphokinase differed greatly among cases during the first week. 43 (46.7%) cases had congenital diseases. 12 (13.0%) cases were reported with abnormal laboratory test results or abnormal signs that are possibly relevant before anesthesia.

**Conclusions:**

In countries that dantrolene is not readily available, early warning, diagnosis, and prompt effective therapies are crucial for MH patients to survive.

**Supplementary Information:**

The online version contains supplementary material available at 10.1186/s12871-021-01328-3.

## Background

Malignant hyperthermia (MH) is a rare but life-threatening pharmacogenetic muscle disorder characterized by abnormal hypermetabolic reactions and commonly triggered in susceptible individuals by volatile anesthetics or succinylcholine, or both. The incidence of MH is estimated between 1/5000 and 1/250000 anesthetics [1–5]. However, the real prevalence of MH susceptibility is very much higher because most people with MH-related genetic mutations never undergo any anesthesias during their lives. Indeed, the predicted genetic prevalence is reported between 1/2000 and 1/3000, and another study reported the prevalence may be as high as 1/400 [6–8]. Malignant hyperthermia mortality reached up to 70% before the introduction of dantrolene [9]. Another study showed the mortality rate was 64% before administration approval of dantrolene [10]. Unfortunately, the specific medicine dantrolene is not readily available in many countries. Due to low incidence, high cost, and short life span, it is quite difficult to get dantrolene when MH episodes happen in the great majority of hospitals in China as well. In China, MH has been often mostly reported in the form of case reports. In the vast majority of cases, dantrolene was not administered. The aim of this study was to find the characteristics of MH under the situation that dantrolene is not readily available.

## Methods

The keyword `malignant hyperthermia` was used to search in Wanfang Database, China National Knowledge Infrastructure, China Science and Technology Journal Database, and China Biology Medicine Database, which are the most commonly used databases in China. Exclusion criteria were dubious MH episodes only caused by Ketamine administration or MH episodes irrelevant to anesthesia.

The MH clinical grading scale (CGS) was used to qualitatively assess the probability of the MH cases. CGS score range, MH rank, and qualitative probability are shown in Table 1. Based on the scoring rule, if more than one indicator represent a single process, only count the indicator with the highest score [11]. For example, both increased creatine kinase (CK) to more than 10,000 IU after anesthetic administration without succinylcholine (15 points) and cola-colored urine after anesthetic administration (10 points) represent the same process: muscle breakdown. Therefore, an individual with the above two abnormal signs and laboratory results would have only 15 points, not 25 points. But if authors offered the ranks or CGS scores, they were directly adopted.

**Table 1:** Clinical grading scale

CGS points	MH rank	MH probability
0	1	MH probability is almost never
3–9	2	MH probability is unlikely
10–19	3	MH probability is somewhat less than likely
20–34	4	MH probability is somewhat greater than likely
35–49	5	MH probability is very likely
50–108	6	MH probability is almost certain

Statistical analysis was performed using SPSS v24 (IBM Corp, Armonk, NY, USA). For continuous variables, for instance, age, maximum end-tidal and arterial CO2, temperature when the patient was first discovered abnormal, etc. in which survival and death groups of variables were compared. Descriptive statistics were expressed as mean (SD) and median (IQR [range]), and independent t-test were used. For categorical variables, for instance, gender, generalized muscular rigidity, cola-colored urine, etc., Pearson’s chi-squared test was used to test the difference between the variables of the two groups by number (proportion). The *P*-value < 0.05 was considered statistically significant.

## Results

Totally 139 relevant articles were retrieved. Dubious MH episodes only caused by Ketamine administration and MH episodes irrelevant to anesthesia were ruled out. The process of identifying the eligible articles is outlined in Supplemental Figure 1. Eventually, 110 articles and 92 cases (85.2% of MH episodes relevant to anesthesia administration reported) were included in the final analysis, but not all data were recorded and reported for these 92 cases [12–121]. Therefore, some variables included less than 92 cases and some patients’ CGS points were underestimated or not estimated. 63 (68.5%) cases were MH rank 6 representing the MH probability is almost certain. 15 (16.3%) cases were MH rank 5 representing the MH probability is very likely. 4 (4.3%) cases were MH rank 4 representing the MH probability somewhat greater than likely.

### Cases sources characteristics and demographics

**Fig. 1:**
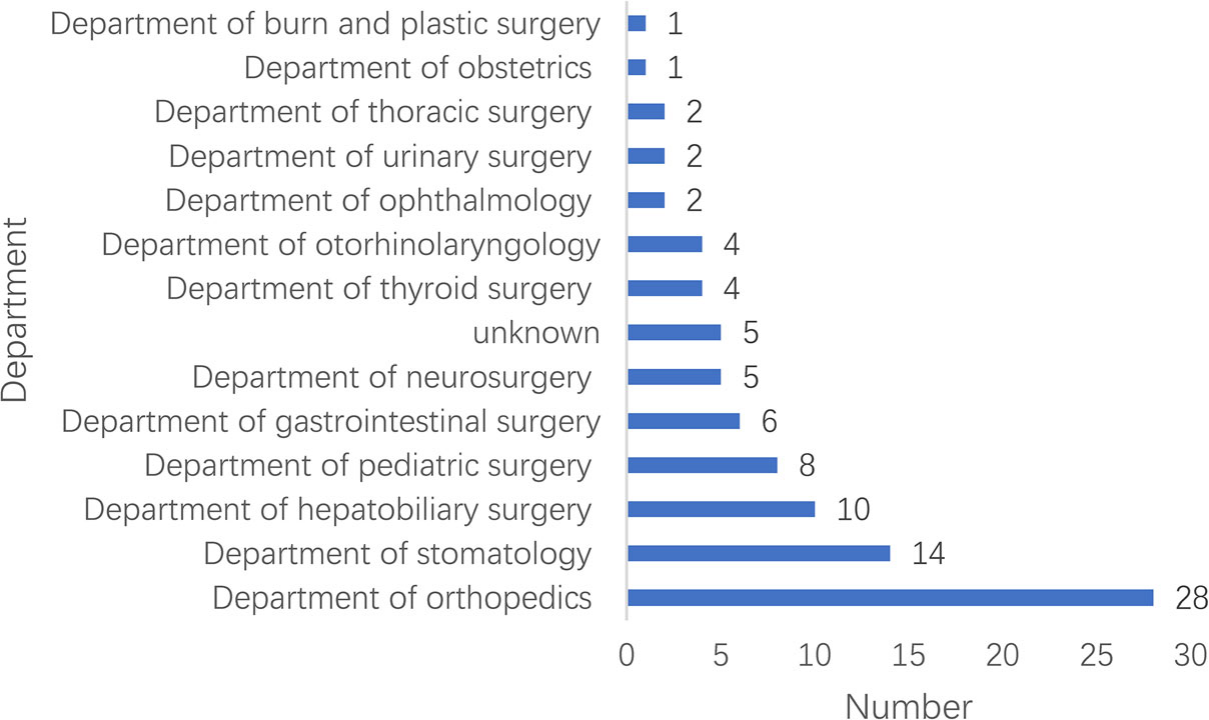
Department distribution of MH cases

**Fig. 2:**
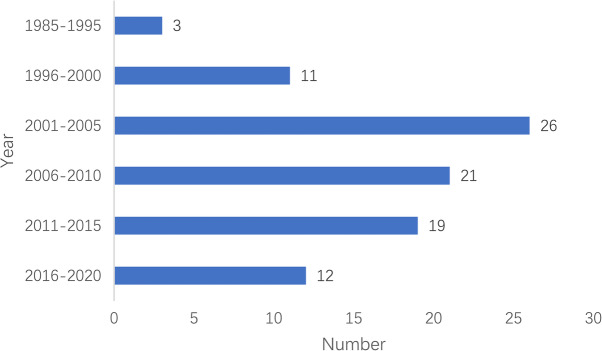
Occurrence year distribution of MH cases

**Fig. 3:**
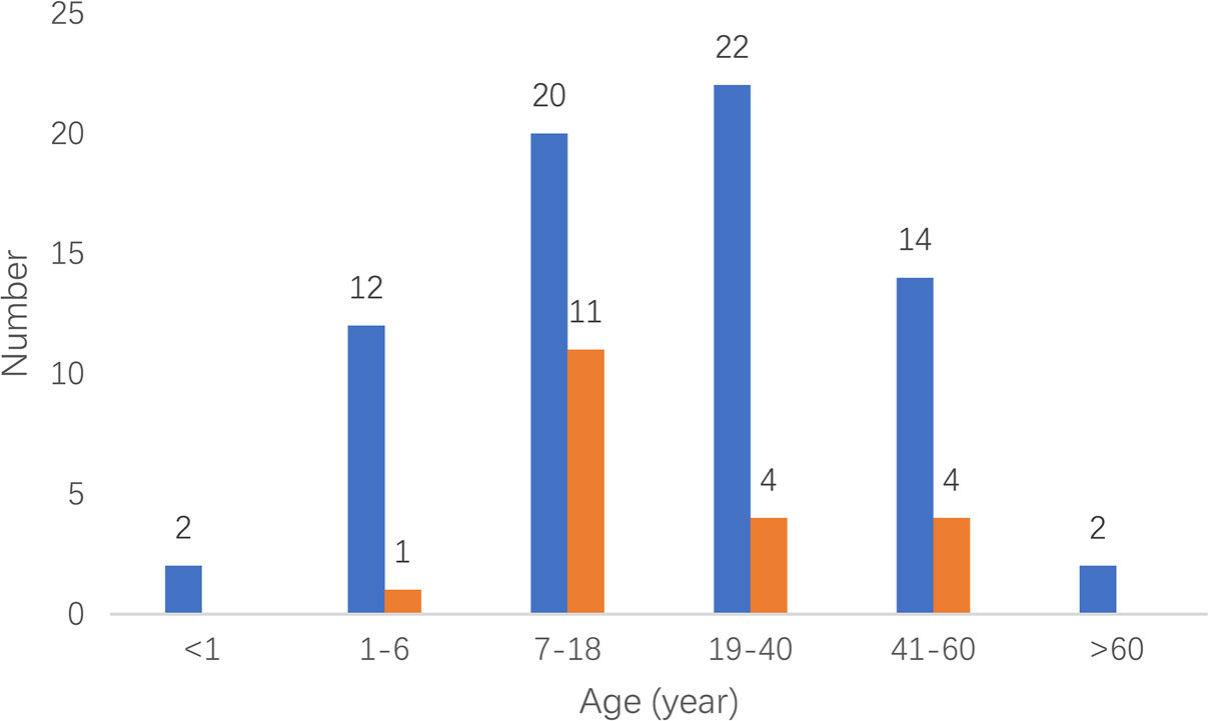
Age distribution of MH cases. Blue, male; red, female

One hundred and ten articles and 92 cases in this study involved 13 departments (Fig. 1) and different years (Fig. 2). The median age was 18.5 (11.8–37.0 [0–70.0]) years. 72 (78.3%) cases were male and 20 (21.7%) cases were female (Fig. 3).

### Outcomes

A total of 50 (54.3%) cases survived and 42 (45.7%) cases died. From 1985 to 2010 the total mortality was 33 (54.1%) cases, whereas the total mortality was down to 9 (29.0%) cases from 2011 to 2020 (Table 2). Compared with the previous phase, the total mortality in the latter phase decreased nearly by half (*P* = 0.023). Of total cases, 8 (8.7%) cases were used dantrolene. Of the 50 survival cases excluding the 8 cases that used dantrolene, there were 29 cases with time data beginning to improve after treatment and the median (IQR [range]) time was 1.0 (0.8–2.0 [0.3–5]) hours.

**Table 2:** Outcome of MH cases. Values are number (proportion)

	Survived	Death
total outcome between 1985 and 2010 (*n* = 61)	28 (45.9%)	33 (54.1%)
total outcome between 2011 and 2019 (*n* = 31)	22 (71.0%)	9 (29.0%)
outcome between 1985 and 2010 without dantrolene (*n* = 59)	26 (44.1%)	33 (55.9%)
outcome between 2011 and 2019 without dantrolene (*n* = 25)	16 (64.0%)	9 (36.0%)

### Anesthetics

Table 3 shows the frequency with which volatile anesthetics or succinylcholine, or both, were administered. Of 76 cases with anesthetics data, five cases used succinylcholine without volatile anesthetic, 17 cases used succinylcholine and volatile anesthetic, and 71 cases only used volatile anesthetic including 32 (45.1%) cases isoflurane, 19 (26.8%) cases used sevoflurane, 18 (25.4%) cases used enflurane, and 2 (2.8%) cases used halothane.

**Table 3:** Anesthetics of MH cases. Values are number (proportion)

Anesthetic drug	Frequency (***n*** = 92)
Succinylcholine +, Volatile -	5 (5.4%)
Succinylcholine +, Volatile +	17 (18.5%)
Succinylcholine -, Volatile +	54 (58.7%)
unrecorded	16 (17.4%)

### The first clinical sign

Of 83 cases with time data from induction of anesthesia to first abnormal sign interval, the median (IQR [range]) time was 1.3 (0.5–2.0 [0–18]) hours. The most frequent initial signs were hypercarbia (31 (33.7%)), sinus tachycardia (23 (25.0%)), hyperthermia (18 (19.6%)), and masseter spasm (10 (10.9%)) (Table 4).

**Table 4:** The first clinical sign of MH cases. Values are number (proportion)

	Frequency (***n*** = 92)
Elevation of end tidal CO_2_	31 (33.7%)
Sinus tachycardia	23 (25.0%)
Rapidly increasing temperature	18 (19.6%)
Masseter spasm	10 (10.9%)
Unrecorded	6 (6.5%)
Reduction of oxygen saturation	4 (4.3%)
Reduction of blood pressure	4 (4.3%)
Convulsion	3 (3.3%)
Elevation of blood pressure	3 (3.3%)
Restlessness	3 (3.3%)
Muscular rigidity	3 (3.3%)
Poor muscle relaxation effectiveness	3 (3.3%)
Dark red blood at surgical field	2 (2.2%)
Elevation of muscular tension	2 (2.2%)
Sweating	2 (2.2%)
Elevation of airway resistance	2 (2.2%)
Neck stiffness	2 (2.2%)
Flushed face	1 (1.1%)
Cyanosis of nail beds	1 (1.1%)
Opisthotonos	1 (1.1%)
Hot soda lime canister	1 (1.1%)
Reduction of heart rate	1 (1.1%)
Depression of ST segment in ECG	1 (1.1%)
Cola-colored urine	1 (1.1%)
Excessive bleeding at surgical field	1 (1.1%)
Muscle tremors	1 (1.1%)

*CO*_*2*_ carbon dioxide; *ECG* electrocardiograph

### Comparisons of survived and death cases

Analysis of the age, gender, history of congenital disease, clinical sign, laboratory result, treatment, and CGS scores between the survived and death cases were as follows (Table 5). Compared with the survived cases, the death cases had higher maximum end-tidal partial pressure of carbon dioxide (PCO_2_) (*P* = 0.033), maximum arterial PCO2 (*P* = 0.006), temperature first measured when the patient was first discovered abnormal (*P* = 0.012), and maximum temperature (*P* < 0.001). Besides, the death cases had less minimum pH (P < 0.001) and higher potassium (P < 0.001) and were more likely to have coagulation disorders (*p* = 0.018). Concerning treatment, cases used furosemide (*P* = 0.024), mannitol (*P* = 0.029), blood purification treatment (*P* = 0.017) had the advantage on the outcome.

**Table 5:** Comparisons of survived and death cases. Values are mean (SD), median (IQR [range]) or number (proportion)

	Survived	Death	***P*** value
Age; y (*n* = 92)	24.2 (16.7)	23.1 (17.3)	0.756
Sex; male (*n* = 92)	40 (80.0%)	32 (76.2%)	0.659
Combined congenital disease (*n* = 92)	21 (42.0%)	22 (52.4%)	0.32
First sign interval; h (*n* = 83)	1.0 (0.2–2.8 [0–11.0])	1.5 (1.0–2.0 [0–18.0])	0.787
Maximum end-tidal PCO_2_; mmHg (*n* = 39)	85.0 (71.8–101.3 [60.0–149.0])	91.0 (86.0–126.5 [75.0–223,0])	0.033
Maximum arterial PCO_2_; mmHg (*n* = 44)	83.0 (73.9–99.4 [53.0–120.0])	101.0 (87.8–152.2 [52.8–250.0])	0.006
T first measured; °C (*n* = 64)	38.5 (38.0–39.1 [35.8–43.0])	39.3 (38.6–41.1 [37.0–42.5])	0.012
Maximum T; °C (*n* = 88)	40.3 (39.3–41.4 [38.3–44.5])	42.3 (42.0–43.1 [39.4–46])	< 0.001
Maximum HR; bpm (*n* = 65)	160.0 (140.0–180.0 [110.0–220.0])	160.0 (150.0–190.0 [120–230.0])	0.187
Generalised muscular rigidity (*n* = 92)	29 (58.0%)	31 (73.8%)	0.113
Normal BP when first discovered (*n* = 92)	18 (36.0%)	8 (19.0%)	0.072
Increased BP when first discovered (*n* = 92)	1 (2.0%)	5 (11.9%)	0.055
No drop in BP when first discovered (*n* = 92)	19 (38.0%)	13 (31.0%)	0.48
Cola-colored urine (*n* = 92)	11 (22.0%)	12 (28.6%)	0.468
Oliguria or anuria (*n* = 92)	9 (18.0%)	13 (31.0%)	0.147
Minimum pH (*n* = 48)	7.14 (7.08–7.22 [6.81–7.40])	6.92 (6.79–7.05 [6.57–7.24])	< 0.001
Potassium; mmol/L (*n* = 44)	5.2 (4.6–5.7 [3.8–6.7])	7.1 (6.5–8.3 [5.7–10.1])	< 0.001
Coagulation disorders (*n* = 92)	11 (22.0%)	19 (45.2%)	0.018
Patient used sodium bicarbonate (*n* = 92)	31 (62.0%)	30 (71.4%)	0.341
Patient used glucocorticoid (*n* = 92)	35 (70.0%)	31 (73.8%)	0.686
Patient used active cooling (*n* = 92)	42 (84.0%)	30 (71.4%)	0.145
Patient used furosemide (*n* = 92)	32 (64.0%)	17 (40.5%)	0.024
Patient used mannitol (*n* = 92)	12 (24.0%)	3 (7.1%)	0.029
Patient used blood purification treatment (*n* = 92)	13 (26.0%)	3 (7.1%)	0.017
Clinical grading scale score; point (*n* = 82)	58.0 (51.0–63.0 [33.0–73.0])	58.0 (51.0–61.0 [33.0–73.0])	0.809

*PCO*_*2*_ partial pressure of carbon dioxide; *T* temperature; *HR* heart rate; *BP* blood pressure

Table 5 Comparisons of survived and death cases. Values are mean (SD), median (IQR [range]) or number (proportion).

### Drug treatment

Of 54 cases with vasoactive drugs data, the most commonly used medications were dopamine (57.4%), epinephrine (53.7%), and norepinephrine (25.9%) (Supplemental Table 1). Besides the vasoactive agents mentioned in Supplemental Table 1 and agents mentioned in Table 5, 11 cases (12.9%) were administered insulin, and 18 cases (19.6%) were administered antibiotics.

### Enzymes

**Fig. 4:**
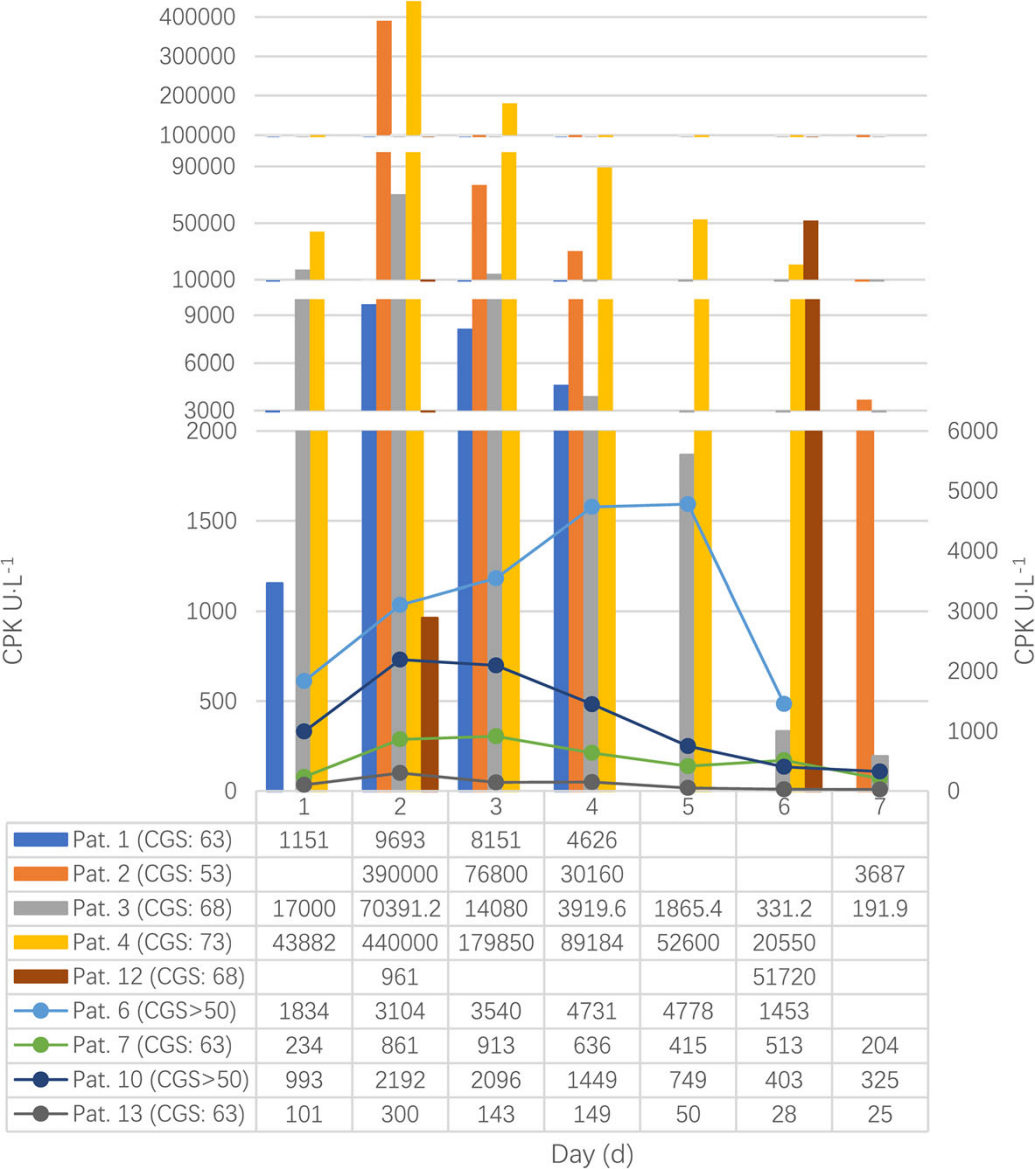
Changes of creatine phosphokinase. CPK, creatine phosphokinase

**Fig. 5:**
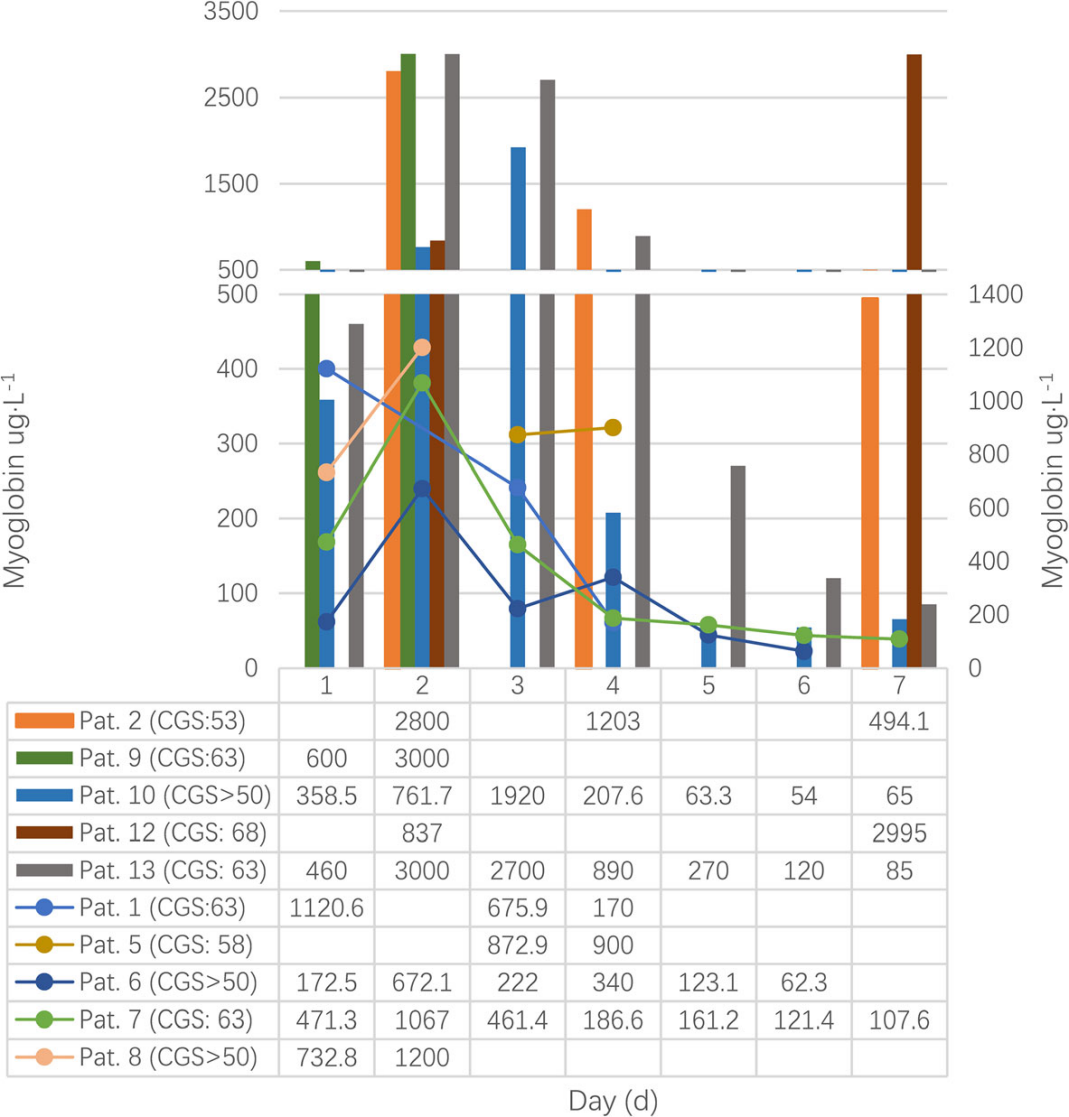
Changes of myoglobin

**Fig. 6:**
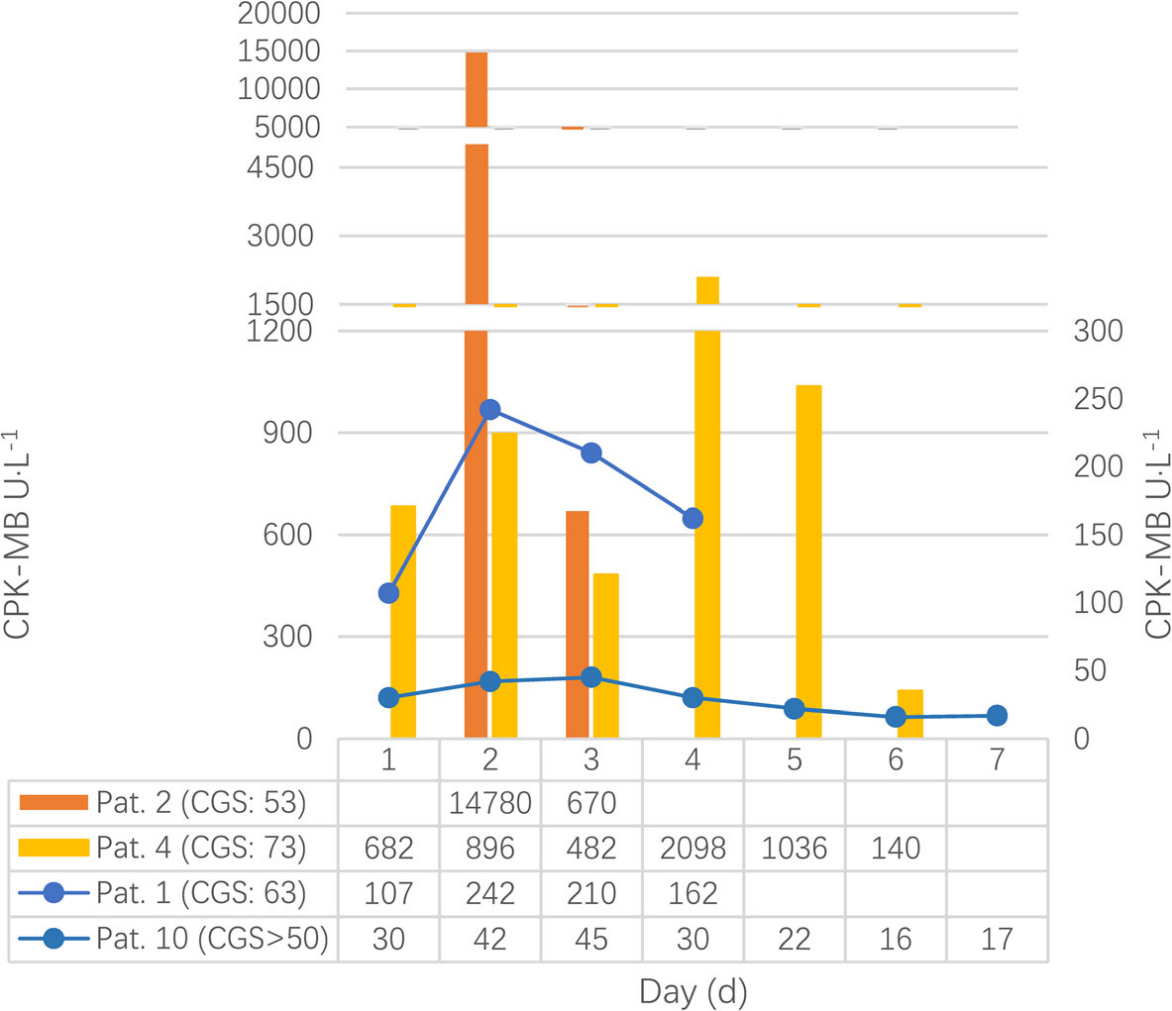
Changes of CPK-MB. CPK-MB, creatine phosphokinase myocardial band

Of total cases, 13 cases were recorded more enzyme data [14, 24, 27, 31, 34, 40, 53, 87, 88, 91, 95, 109, 116]. The CGS score of the patient six, eight and ten were graded by original authors. As Figs. 4, 5 and 6 shown, creatine phosphokinase (CPK), myoglobin, and creatine phosphokinase myocardial band (CPK-MB) varied greatly during the first week, and there were significant differences among these patients as well.

### History of congenital disease and abnormal characteristics before anesthesia

43 (46.7%) cases had congenital diseases. 12 (13.0%) cases were reported with abnormal laboratory test results or abnormal signs that are possibly relevant before anesthesia. Among these cases, 6 (6.5%) cases were with increased CPK, 4 (4.3%) cases with increased alkaline phosphatase (ALP), 2 (2.2%) cases with increased CPK-MB,1 (1.1%) cases with increased lactic dehydrogenase (LDH), and 3 (3.3%) cases were recorded with a mildly elevated body temperature of unknown origin.

### Diagnostic testing

**Fig. 7:**
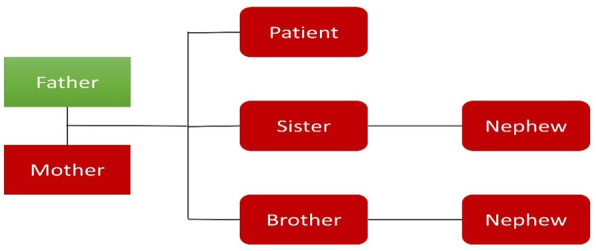
RYR1 testing result in one family

Of the total cases, 7 (7.6%) cases took relevant examinations and showed positive results. In three cases, the muscles of the patients were soaked in succinylcholine solutions and all of them tested positive and contracted strongly. Muscle biopsy was performed in four cases, among which one case showed hyaline degeneration in quadriceps femoris, one case with vacuolar degeneration and myolysis in quadriceps femoris, one case with severe vacuolar degeneration in striated muscle, and one case with inflammatory and degeneration in gastrocnemius muscle. In another case, as Fig. 7 shown, seven members of the immediate family of the patient took the genetic testing and six members in red were tested positive and have MH susceptibility [45].

## Discussion

Totally 110 articles and 92 cases were used from the most commonly used databases in China. Exclusion criteria were dubious MH episodes only caused by Ketamine administration or MH episodes irrelevant to anesthesia. This study may be limited by incomplete patient data and underreporting, but analysis bias seems to be minimal because there were no significant differences between comparisons of survived and death cases.

For the incident departments, they were concentrated in departments of orthopedics, stomatology, and hepatobiliary surgery. Around half of the incident years focused on 2001–2010. The male to female of MH cases was 3.5:1. More than half of MH cases focused on the 7–18 and 19–40 demographic. In all these MH cases reported, the total mortality was 42 (45.7%), less than the mortality rate 64–70% reported before administration of dantrolene [9, 10]. Even in the absence of dantrolene, the mortality was down to 36.0% from 2011 to 2020. In terms of anesthetics, more than half of all these cases were administered volatile anesthetic without succinylcholine, mainly including isoflurane, sevoflurane, and enflurane. Besides, the most frequent initial signs of these cases were hypercarbia, sinus tachycardia, hyperthermia, and masseter spasm.

Although there were no significant differences between comparisons of survived and death cases, some clues were still found from the analysis. From the comparisons, the death cases had higher maximum end-tidal PCO_2_, maximum arterial PCO_2_, temperature first measured when the patient was first discovered abnormal, maximum temperature and potassium, and had more serious metabolic acidosis and more possibility of coagulation disorders. On the treatment side, cases that used furosemide, mannitol, blood purification treatment had a significant advantage on the outcome, which showed renoprotective therapies play important roles in outcomes in these MH cases.

The 13 cases with more enzyme data were all at MH rank 6. But there were wide differences in concentration of CPK, myoglobin, and CPK-MB between these `almost certain` cases. Therefore, the low size of these enzyme value might be that they can’t be used to rule out MH episode or determine the severity of MH, which confirm the study made by Carpenter et al. [122] that different RYR1 variants vary in the severity of CPK concentration. Besides, most of the cases’ pick time was on the second day, while occasional cases were on the third, fifth, or sixth day.

Almost half of these MH cases had congenital diseases. Around one in eight of the cases had abnormal enzyme results and mildly elevated body temperature. Therefore, anesthesiologists should take precautions when there are congenital diseases, these abnormal enzyme results or abnormally elevated body temperature for unexplained reason in pre-anesthesia patients and need to avoid administering volatile anesthetics and depolarizing neuromuscular blocking drugs muscle relaxants and strengthen monitoring in the susceptible individuals.

MH is inherited as an autosomal dominant disorder. Seven members of the immediate family of one patient all took the genetic testing, and except for the patient’s father the other six members all tested positive and have MH susceptibility. Therefore, once MH episode happens, all family members later need to be advised to take genetic testing, and if the test is positive they are further advised to make warning cards, bracelets, or necklaces with MH susceptible on them and carry them at all times to alert anesthesiologist, nurse anesthetists, and relevant staffs in case they need anesthesia in the future.

MH is a rare but life-threatening disorder. When body temperature is over 41 °C, disseminated intravascular coagulation (DIC) is the most common cause of death [1]. The possibility of any complication almost triples per two degrees Celsius rise in maximum body temperature [123]. The lack of dantrolene is the main limitation of MH treatment. Therefore, early warning and diagnosis and prompt effective therapies are crucial for MH patients to survive, especially in the countries that dantrolene is not readily available. There is a pressing need to establish an MH website and a telephone hotline available around the clock in China and countries that have not had these yet, and anesthesiologists, nurse anesthetists, and relevant staff are also urged to register MH episodes by real-name or anonymity. All information can be collected through the internet and directly uploaded to the national database in real-time. With the consent of those MH susceptible people, the identity information is uploaded. And the information can only be disclosed in internal systems among hospitals and related units. Once these people need to undergo anesthesia, anesthesiologists, nurse anesthetists, and relevant staff can receive alerts immediately. Besides, the need to carry out extensive publicity and education concerning MH incidence, clinical presentation, pathophysiology, diagnosis, and treatment is also urgent, not only on professionals and also ordinary people. Let as many people as possible realize the importance and seriousness. MH susceptible persons would volunteer to upload their identity information by themselves.

In conclusion, in countries that dantrolene is not readily available, early warning, diagnosis, and prompt effective therapies are crucial for MH patients to survive.

## Supplementary Information

**Additional file 1: Supplemental Figure S1.** Flow chart of the study selection procedure. MH, malignant hyperthermia.

**Additional file 2: Supplemental Table S1.** The use of vasoactive agents of Malignant hyperthermia cases.

## Data Availability

The datasets used and analysed during the current study are available from the author on request.

## Abbreviations

IQR: Interquartile range
MH: Malignant hyperthermia
CGS: Clinical grading scale
CK: Creatine kinase
SD: Standard deviation
CO_2_: Carbon dioxide
ECG: Electrocardiograph
PCO_2_: Partial pressure of carbon dioxide
T: Temperature
HR: Heart rate
BP: Blood pressure
CPK: Creatine phosphokinase
CPK-MB: Creatine phosphokinase myocardial band
ALP: Alkaline phosphatase
LDH: Lactic dehydrogenase
DIC: Disseminated intravascular coagulation
