# Supplementary figures and images for: Malignant hyperthermia when dantrolene is not readily available
Source: BMC Anesthesiol. 2021 Apr 16;21:119. doi: 10.1186/s12871-021-01328-3 (PMC8051048; doi:10.1186/s12871-021-01328-3)

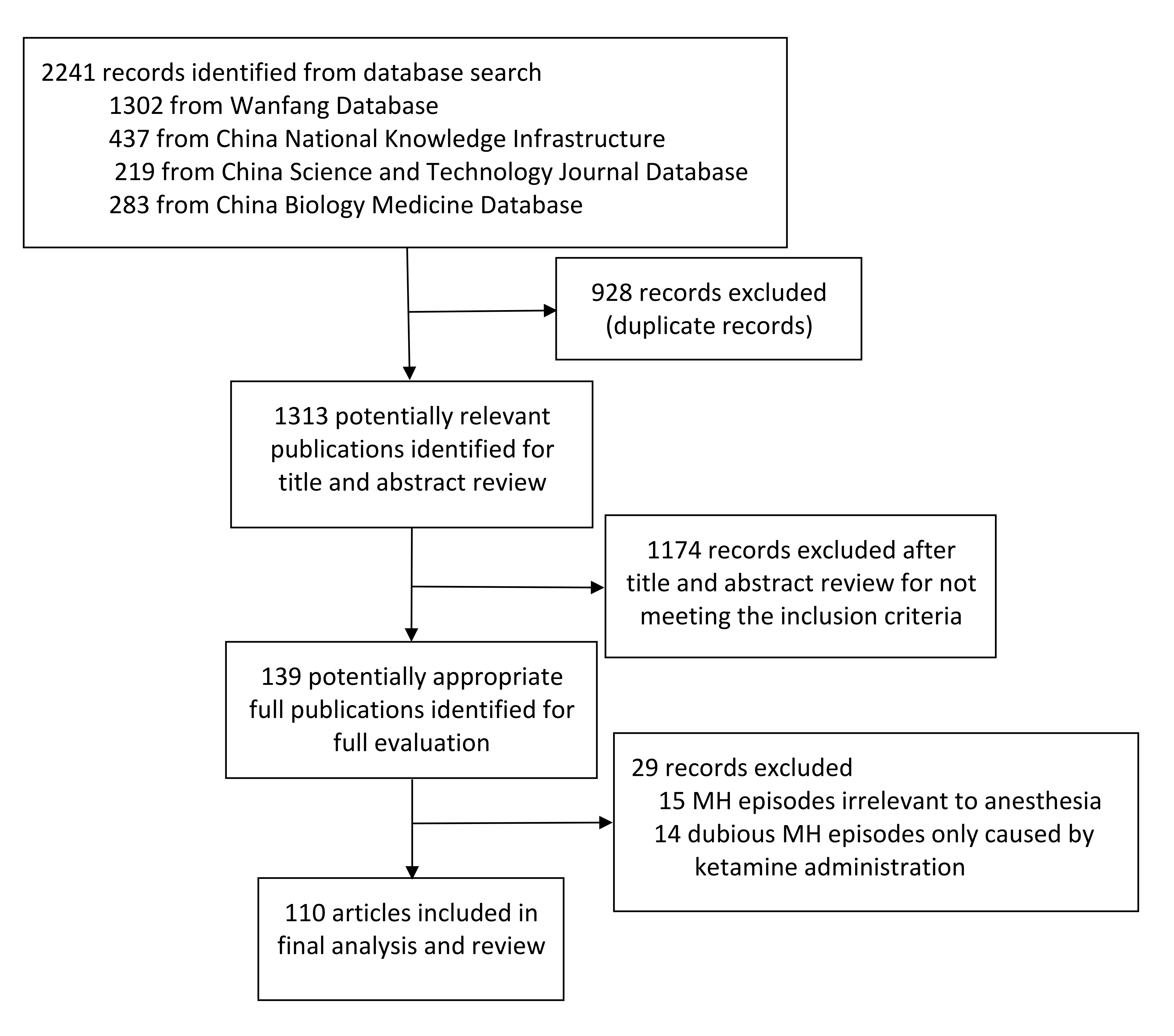

Supplement: Supplementary file 1 — Additional file 1: Supplemental Figure S1. Flow chart of the study selection procedure. MH, malignant hyperthermia. [file 12871_2021_1328_MOESM1_ESM.tif]
