# Supplementary material for: Malignant hyperthermia when dantrolene is not readily available
Source: BMC Anesthesiol. 2021 Apr 16;21:119. doi: 10.1186/s12871-021-01328-3 (PMC8051048; doi:10.1186/s12871-021-01328-3)
Supplement: Supplementary file 2 — Additional file 2: Supplemental Table S1. The use of vasoactive agents of Malignant hyperthermia cases. [file 12871_2021_1328_MOESM2_ESM.docx]

Supplemental Table 1 The use of vasoactive agents of MH cases

|  | **Frequency, n/total (%)** |
| --- | --- |
| Dopamine | 31/54 (57.4) |
| Epinephrine | 29/54 (53.7) |
| Norepinephrine | 14/54 (25.9) |
| Esmolol | 10/54 (18.5) |
| Lidocaine | 10/54 (18.5) |
| Ephedrine | 8/54 (14.8) |
| Atropine | 6/54 (11.1) |
| Calcium gluconate | 6/54 (11.1) |
| Cedilanid | 5/54 (9.3) |
| Phenylephrine | 5/54 (9.3) |
| Isoproterenol | 5/54 (9.3) |
| Dobutamine | 3/54 (5.6) |
| Aminophylline | 2/54 (3.7) |
| Procainamide | 2/54 (3.7) |
| Amiodarone | 1/54 (1.9) |
| Propafenone | 1/54 (1.9) |
| Metaraminol | 1/54 (1.9) |
